# Supplementary material for: Learning curve and surgical outcome of robotic assisted colorectal surgery with ERAS program
Source: Sci Rep. 2022 Nov 29;12:20566. doi: 10.1038/s41598-022-24665-w (PMC9709162; doi:10.1038/s41598-022-24665-w)
Supplement: Supplementary file 1 — Supplementary Information 1. [file 41598_2022_24665_MOESM1_ESM.zip › LSC Raw data-Ñ[▒K/136/H_ñw╝╨░O▒Kñσ.pdf]

正式摘要(電子簽章：000000224663218)

W52 / W52 ☐ 英文標題(注意:無英文姓名-請補病人基本檔!) ☐【讀取】憑證

臺中榮民總醫院

出院病歷摘要

轉入號： 轉出號：

[redacted]

(8)流水編號	
(9)入院日期	110/08/31 (2021) 直腸外科 W52 - 111
(10)轉科日期	
(11)出院日期	110/09/09 (2021) 住院天數計 9 日

診斷

- 入院：1. Sigmoid colon adenocarcinoma s/p laparoscopic anterior resection and anastomosis on 2021/09/01
  -pT3N1aM1a(Liver, mesocolic soft tissue, mesocolic lymph nodes(1/20))
  [Past history]
  1. Adenocarcinoma of lung, RML, EGFR L858R, cT2aN0M0, s/p thoracoscopic lobectomy of lung, RML with LNs dissection. on 2019/03/25, pT2aN0M0, stage IB, recurrence with pleural seeding and malignant pleural effusion, stage IVA, ECOG PS = 1
  - s/p Afatinib 40 mg (2020/10/05-19), 30 mg(10/19-01/24)
  - shift to Tarceva (2021/01/25) due to intolerance paronychia
  2. Right toe cellulitis, s/p Oxacillin (2020/09/27-)
  3. Prostate cancer, with multiple metastasis to bone and liver, cT4NxM1, under hormone therapy with casadex and Firmagon(2019/01/15~).
  4. Left side hydronephrosis,s/p D-J placement at Tung's hospital, s/p PCN placement on 2019/01/28, s/p D-J removal on 2019/01/30.
  5. CRAO, under caduet and bokey
- 出院：Sigmoid colon adenocarcinoma s/p laparoscopic anterior resection and anastomosis on 2021/09/01
  pT3N1a(1/23)M1a(liver), MD, AI+, PI+, TuDp+:8, CRM-:4mm, DisM:-:5cm, pMMR stage IVA
  1. Adenocarcinoma of lung, RML, EGFR L858R, cT2aN0M0, s/p thoracoscopic lobectomy of lung, RML with LNs dissection. on 2019/03/25, pT2aN0M0, stage IB, recurrence with pleural seeding and malignant pleural effusion, stage IVA, ECOG PS = 1
  - s/p Afatinib 40 mg (2020/10/05-19), 30 mg(10/19-01/24)
  - shift to Tarceva (2021/01/25) due to intolerance paronychia
  2. Right toe cellulitis, s/p Oxacillin (2020/09/27-)
  3. Prostate cancer, with multiple metastasis to bone and liver, cT4NxM1, under hormone therapy with casadex and Firmagon(2019/01/15~).
  4. Left side hydronephrosis,s/p D-J placement at Tung's hospital, s/p PCN placement on 2019/01/28, s/p D-J removal on 2019/01/30.
  5. CRAO, under caduet and bokey

主訴

- For scheduled surgery

病史

- This is a 77y/o male had past history of
  1. Adenocarcinoma of lung, RML, EGFR L858R, cT2aN0M0, s/p thoracoscopic lobectomy of lung, RML with LNs dissection. on 2019/03/25, pT2aN0M0, stage IB, recurrence with pleural seeding and malignant pleural effusion, stage IVA, ECOG PS = 1
  - s/p Afatinib 40 mg (2020/10/05-19), 30 mg(10/19-01/24)
  - shift to Tarceva (2021/01/25) due to intolerance paronychia
  2. Right toe cellulitis, s/p Oxacillin (2020/09/27-)
  3. Prostate cancer, with multiple metastasis to bone and liver, cT4NxM1, under hormone therapy with casadex and Firmagon(2019/01/15~).
  4. Left side hydronephrosis,s/p D-J placement at Tung's hospital, s/p PCN placement on 2019/01/28, s/p D-J removal on 2019/01/30.
  5. CRAO, under caduet and bokey
  Smoking history 0.5 PPD for 10 years, quit for more than 30 years. His ADL is totally independent.

- He had bloody stool with conspitation since half years ago. The CEA followed in our hospital showed gradually increased, final data on 2021/07/17 was 64.74. Colonoscopy on 2021/08/02 showed sigmoid tumor with partial obstruction. He was admitted this time for surgery.

體檢發現

-
  - Performance status(ECOG)： 0
  - General appearance: A well-nourished person, with chronic ill looking. Consciousness: Alert Pupil size: 3-3 mm Light reflex:
    - Normal light reflex

    Vital signs:

date	體溫(BT)	脈搏(P)	呼吸(R)	血壓(BP)	身高(BH)	體重(BW)	BMI
20210831	36.7℃	83次/分	18次/分	169/90mmHg	159.6cm	69.7kg	27.4
  - Skin
    - Normal skin turgor , No jaundice , No cyanosis , No rash , No wound / ulcer / scar
  - Lymphadenopathy
    - No lymphadenopathy
  - HEENT
    - Head: No shape deformity , No hair change , Normal lip
    - Eyes: ＊Conjunctiva: Normal , ＊Sclera: Not icteric , ＊Cornea: Clear , ＊Eyelids: Normal
    - Ears: No deformity
    - Nose: Normal appearance
    - Mouth and throat: No oral mucosa change , No throat injection , No tonsil enlargement
  - Neck
    - No thyroid enlargement , Neck: Jugular vein
  - Chest
    - Inspection: Normal and symmetric respiratory movement , No deformity , Normal breast
    - Palpation: No palpable mass , No tender point
    - Auscultation: No rales , No rhonchi , No wheezing , Chest / Auscultation / Stridor No stridor , No peripheral brochial sound , No friction rub
  - Heart
    - Inspection: No visible apical impulse
    - Palpation: Not palpable PMI , No thrill , No heave
    - Auscultation: Regular heart beats , No S3 , No S4 , No murmur
  - Abdomen
    - Inspection: No distension , No striae
    - Auscultation: Normal bowel sound ,
    - Palpation: No hepatomegaly , No tenderness , No rebounding pain , No palpable mass , No splenomegaly
    - Percussion:
  - Genito-anus patient refuse
  - Back and spine
    - No deformity , No knocking pain
  - Extremities
    - No deformity , No clubbing finger , No pitting edema , Normal joint , Normal peripheral pulse
  - Neurological examination
    - No mental status change
    - Normal cranial nerve
    - Normal meninges
    - flexor response
    - Normal motor
    - Normal sensory
    - Normal cerebellum
    - Normal Stance & Gait

手術日期及方法

-

- 手術日期及方法20210901 1. Laparoscopic anterior resection with end-to-side anastomosis, endo-GIA 60/4.1mm*1, ILS 29mm, re-inforcement with silk: nil, bilateral doughnuts: intact, air leak test: done, no air leak.
  2. High ligation (-): IMA below left colic artery.
  3. Cut margin: proximal>10cm, distal 5cm.
  4. Reperitonealization: nil.
  5. Take down of splenic flexure: yes.
  6. One 12Fr. Penrose drain at pelvis.
  7. Liver S2 excisional biospy

住院治療經過

- After admission, the patient received laparoscopic anterior resection and anastomosis on 2021/09/01 smoothly. He tolerated the operation well. We gradually removed NG and started to try diet with adequate digestion. KRAS and BRAF mutation test are pending. Hemovac and wound stitches were removed, and he discharged under stable condition. OPD follow up was arranged.

合併症

一般檢查

- 累積報告 :

DATE	TIME	ALB	TP	BIL.T	BIL.D	ALKP	AST	ALT	LDH	AMY	LIPASE	NH3	Ferritin	UA
1100901	1417			0.42	0.12		26	16						
1100906	0934			0.40	0.14		14	5						

DATE	TIME	WBC	RBC	HGB	HCT	MCV	PLT	BLST	NEUT	BAND	SEG	LYM	MON	EOS	BAS
1100901	1421	11940		11.1	33.0		218		92.4			4.3	3.1	0.1	0.1
1100906	0912	4960		11.1			257		60.6			20.4	10.1	8.3	0.6

DATE	TIME	NA	K	CL	BUN	CREAT	eGFR	CA	Mg	P	CRP	ESR	PCT	D-Dimer	LACTATE
1100901	1417	141	3.1		11	0.80	99.63								
1100906	0934	136	4.0		14	0.89	88.1	8.0							

DATE	TIME	GLUCOSE	HbA1C	CHOL	TG	HDL	LDL	KETONE	CK	CKMB	TRO-T	PT-P	APTT-P	NT-pro BNP
1100831	1923		5.6											

- 放射線報告 :**檢驗項目 :CT SCAN,CHEST, HILA, TRACHEA 報告日期 :1100827**

  主　訴 :
  Result:Y
  Finding:
  Chest CT with IV contrast administration:
  1. A case of lung CA, RML, s/p op for follow up.
  2. Post operative fibrotic change over right lung.
  3. Stable in size of the nodularity over right apical pleura but increasing in size of the metastatic nodules over residual lungs as compared with previous CT on 20210503.
  4. No enlarged LNs over mediastinum and bil. hili region.
  5. Presence of faint osteoblastic lesion at T12 vertebral body. Relatively stable in condition.
  6. Normal visible pancreas, spleen and bil. adrenal glands.
  7. Atherosclerotic change with calcification of aorta and coronary arteries.
  8. Hepatic cysts over both lobes of liver. Presence of several poor enhancing mass lesions less than 33mm in size over both lobes of liver, the possibility of liver metastasis should be considered.
  9. Presence of focal wall thickening at the gallbladder, the possibility of adenomyomatosis cannot be ruled out.
  10. Bilateral renal cysts.

  Impression:
  Lung CA, RML, s/p op with progressive lung and liver metastasis

特殊檢查

病理報告

- 活體組織切片病理報告　手術日期：**110/08/05**　病理號：**S1024041**

診斷醫師:孫志豪 報告日期:110/08/09
Pathologic diagnosis:
Sigmoid colon, 20 cm from anal verge, labeled "tumor", endoscopic biopsy --- Adenocarcinoma, moderately differentiated.

- Gross description:
  The specimen consists of 2 pieces of tan soft tissue, up to 0.4x0.3x0.2 cm. All for section.
  Microscopic description:
  Section shows colon mucosa with invasive moderately differentiated carcinoma exhibiting glandular differentiation.
  Comment:
  Further clinicopathological correlation and study are suggested.
  This case has been peer reviewed by two doctors.
  #T-59300_2 #M-81403_2 2 1340 00000F

活體組織切片病理報告 手術日期：**110/09/01** 病理號：**S1027333**

診斷醫師:陳志榮 報告日期:110/09/07
Pathologic diagnosis:
1. Liver, S2, hepatectomy --- Consistent with metastatic colonic adenocarcinoma, moderately differentiated. Resection margin cannot be evaluated due to fragmented specimen.
2. Sigmoid colon, laparoscopic anterior resection --- Adenocarcinoma, moderately differentiated, with extracellular mucin production. Tumor invaded mesocolic soft tissue with tumor deposits and mesocolic lymph nodes metastasis (1/20). The bilateral cut ends and radial margin are free of tumor.
3. IMA root, dissection --- Unremarkable lymph nodes (0/3).
4. Colon, cut end, distal, resection --- Unremarkable.
5. "Lymph node", mesentary, dissection --- Metastatic adenocarcinoma, moderately differentiated, in soft tissue. No lymph node seen.

Ancillary study for diagnosis:
1. IHC stains for CDX2 (+) and TTF-1 specific(-) done for section G.
2. IHC stains for PMS2 and MSH6 are done on section G. All above markers are preserved.

Prognostic and predictive factor:
1. Tumor size: 4 cm.
2. Depth of invasion: Mesocolic soft tissue.
3. Histologic grade: G2: Moderately differentiated.
4. Angiolymphatic invasion: Present.
5. Perineural invasion: Present.
6. Tumor deposits (discontinuous extramural extension): Present, specify number of deposits: 8.
7. Macroscopic tumor perforation: Not identified.
8. Surgical Margins: a) Proximal Margin: Uninvolved by invasive carcinoma, distance of tumor from margin: 17 cm. b) Distal Margin: Uninvolved by invasive carcinoma, distance of tumor from margin: 5 cm. c) Radial or Mesenteric Margin: Uninvolved by invasive carcinoma, distance of tumor from margin: 4 cm.
9. Lymph node status: Involved (1/23).
10. Extranodal involvement: Not identified.
11. Tumor regression grading S/P CCRT: No known presurgical therapy.
12. Type of polyp in which invasive carcinoma arose: Not identified.
13. Additional pathologic findings: Not identified.
14. Pathological TNM Stage: pT3N1aM1a(According to the eighth edition, American Joint Committee on Cancer Staging Guidelines for Tumors).
15. TNM descriptors: Not applicable.

Gross description:
The specimen consists of 1) "liver S2", multiple pieces of tan soft tissue, up to 0.6x0.6x0.6 cm. 2) a segment of colon, 25 cm in length and 5 cm in proximal cut end circumference, 6 cm in distal circumference, and mesocolic soft tissue. A gray tan firm annular elevated and centrally ulcerative tumor, 4x3 cm, is noted, 5 cm to the distal cut end. On cross section, the tumor invades to the mesocolic soft tissue with 4 cm to the radial margin, and serosal retraction is also noted. The both cut ends are free of tumor grossly. 3) IMA root, a piece of tan yellow soft tissue, 3.5x2x1 cm. 4) distal cut end, a piece of tan mucosa-covered soft tissue, 2.5x2x1.5 cm. 5) "mesentary LN", a piece of tan yellow soft tissue, 2x2x1 cm. Representative part for section: A) specimen 1 B) specimen 3 C) specimen 4 D) specimen 5 (bisected) E)proximal cut end F) radial cut end G-K) tumor L) soft tissue lesion M-P) lymph nodes.

- Note:
  1. The interpretation of MMR panel and recommended studies are as below: (1) No loss of nuclear expression of MMR proteins: low probability of microsatellite instability-high (MSI-H) . (2) Loss of nuclear expression of MLH1 and PMS2: testing for methylation of the MLH1 promoter and/or mutation of BRAF is indicated (the presence of a BRAF V600E mutation and/or MLH1 methylation suggests that the tumor is sporadic and germline evaluation is probably not indicated; absence of both MLH1 methylation and of BRAF V600E mutation suggests the possibility of Lynch syndrome, and sequencing and/or large deletion/duplication testing of germline MLH1 may be indicated). (3) Loss of nuclear expression of MSH2 and MSH6: high probability of Lynch syndrome (sequencing and/or large deletion/duplication testing of germline MSH2 may be indicated, and, if negative, sequencing and/or large deletion/duplication testing of germline MSH6 may be indicated). (4) Loss of nuclear expression of MSH6 only: high probability of Lynch syndrome (sequencing and/or large deletion/duplication testing of germline MSH6 may be indicated). (5) Loss of nuclear expression of PMS2 only: high probability of Lynch syndrome (sequencing and/or large deletion/duplication testing of germline PMS2 may be indicated). (6) Please note: There are exceptions to the above IHC interpretations. These results should not be considered in isolation, and clinical correlation with genetic counseling is recommended to assess the need for germline testing.
  2. This case has been peer reviewed by two doctors.
  #T-59471_2 #M-81403_2 2 1105 S0000F
  #T-D452B_2 #M-81406_2 2 1105 000000
  #T-C4560_2 #M-81406_2 2 1105 000000

其它

出院時情況

- 3.改門診治療

出院指示

藥名	劑量	劑量單位	用法	頻次	數量	數量單位	現況	天數	單位
Voren-K tab 25mg	1	TAB	PO	TID	15	TAB	TH	5	天
(□)Takepron OD tab 30mg	1	TAB	PO	QDAC	5	TAB	TH	5	天
Ultracet tab	1	TAB	PO	Q8H	15	TAB	TH	5	天

出院計劃

- 門診日期
  - date on 2021/09/13

主治醫師 2222H 林俊余 / 住院醫師 9739F 趙雅芸

正式摘要(電子簽章：000000224663218)

W52 / W52

doctorRank:B
